# Cytological Evidence of Telocyte Involvement in Skin Immune Regulation Following Jet Needle-Free Injection of an Inactivated Porcine Circovirus Vaccine

**DOI:** 10.3390/vetsci13050467

**Published:** 2026-05-12

**Authors:** Haiyan Wang, Chunyuan Dai, Mingfa Yang, Jiasen Feng, Xiangfei Meng, Zhaoxuan Zhu, Xinzi Guo, Ping Yang, Yu Lu

**Affiliations:** 1 College of Veterinary Medicine, Nanjing Agricultural University, No. 1 Weigang, Nanjing 210095, China; wang-haiyan1983@163.com (H.W.); daichunyuan@stu.njau.edu.cn (C.D.); fengjiasen@stu.njau.edu.cn (J.F.); 2020107023@stu.njau.edu.cn (X.M.); zhaoxuanzhu@njau.edu.cn (Z.Z.); 15121225@stu.njau.edu.cn (X.G.); 2 GuoTai (Taizhou) Center of Technology Innovation for Veterinary Biologicals, Taizhou 225300, China; 3 National Research Center of Engineering and Technology for Veterinary Biologicals, Institute of Veterinary Immunology and Engineering, Jiangsu Academy of Agricultural Sciences, Nanjing210014, China; 4 State Key Laboratory for Animal Disease Control and Prevention, Harbin Veterinary Research Institute of Chinese Academy of Agricultural Sciences, Harbin 150069, China; yangmingfa@caas.cn

**Keywords:** pig, porcine circovirus vaccine, telocytes, jet needle-free injection, histopathological changes, ultrastructural changes

## Abstract

A recently identified mesenchymal cell population, telocytes (TCs), has been found in many tissues of different animal species. Jet needle-free injection (JNFI) is a promising non-invasive drug-delivery method that can trigger effective immune responses in the skin. In this preliminary morphological study, an inactivated porcine circovirus vaccine was delivered into pig neck skin by JNFI, and untreated normal neck skin served as the control. Histopathology, immunohistochemistry (IHC), immunofluorescence (IF), and transmission electron microscopy (TEM) were used to evaluate TC distribution, ultrastructure, and selected quantitative TEM parameters 24 h after injection. TCs were widely distributed in porcine skin, located between collagen fibers and around blood vessels, adipocytes, and sweat glands. They were also observed in contact with mast cells. TCs around sweat glands were CD34^+^, Vimentin^+^, and α-SMA^+^, whereas TCs at other sites were CD34^+^, Vimentin^+^, and α-SMA^−^. After JNFI, inflammatory cell infiltration into the skin was observed; TCs were in contact with these cells, and TCs surrounding adipocytes redistributed into the adjacent loose connective tissue. Quantitative TEM analysis showed that TC profile density and visible telopod length did not differ significantly between normal skin and JNFI 24 h skin (*p* ≥ 0.05). In contrast, vesicle profiles per TC increased significantly in both perivascular and adipose-associated compartments (*p* < 0.05). These findings provide morphological evidence suggesting that TCs may participate in early cutaneous responses after JNFI vaccine delivery.

## Simple Summary

Telocytes are a special type of connective tissue cell found in many organs, but their role in skin immune responses is still unclear. Needle-free jet injection is a promising way to deliver vaccines through the skin, yet little is known about how skin support cells respond to this process. In this preliminary study, an inactivated porcine circovirus vaccine was delivered into pig skin by needle-free jet injection, and the distribution, ultrastructure, and selected quantitative transmission electron microscopy features of telocytes were examined 24 h later. Telocytes were widely present in pig skin and were found around collagen fibers, blood vessels, sweat glands, and fat cells. After injection, inflammatory cells entered the skin, and telocytes were often seen in close contact with them. Quantitative transmission electron microscopy analysis showed that telocyte-associated vesicle profiles increased significantly after injection (*p* < 0.05), whereas telocyte profile density and visible telopod length did not differ significantly. These findings suggest that telocytes may participate in early local skin responses after needle-free injection. The work provides morphological evidence that can guide future functional studies of cutaneous vaccine delivery.

## 1. Introduction

Skin serves as a protective barrier exposed to numerous potential pathogenic organisms. When healthy and undamaged, skin envelops the body and provides a physical barrier, preventing pathogens from entering the body. Consistent with other organs, skin-invading pathogens are detected by pattern-recognition receptors (PRRs) [1]. Consequently, skin possesses two primary roles: serving as a physical barrier and facilitating immune reactions. The skin immune system consists of two components—innate immunity and adaptive immunity—involving various immune cells in both the epidermis and the dermis [2]. Langerhans cells and keratinocytes within the epidermis function as the primary line of immune defense, possessing the capabilities to recognize and process antigens. Meanwhile, the dermal layer harbors rich populations of immune cells, including mast cells, macrophages, dendritic cells, and T cells [3]. The dermal layer represents the principal location for immune responses, housing double the quantity of immune cells compared to those found in the circulatory system [4]. These immune cells are not randomly distributed within the dermal architecture, but are instead organized in association with specific anatomical compartments of the skin [5]. The cutaneous immune system comprises various specialized immune cell subsets, including Langerhans cells and T lymphocytes in the epidermis, as well as αβ T cells, γδ T cells, natural killer (NK) cells, dendritic cells, B cells, mast cells, and macrophages in the dermis. Together, these cells form a complex skin immune network [6]. For example, innate immune cells residing in the skin, including mast cells and dendritic cells, work cooperatively with T cells and keratinocytes to initiate and drive immune responses [7]. Dermal fibroblasts not only participate in tissue repair but also regulate local immune responses through the secretion of cytokines and chemokines [8]. During the wound-healing process, the coordinated activities of immune and non-immune cells are crucial for pathogen clearance, the removal of necrotic tissue, and the guidance of damaged tissue regeneration [9].

Telocytes (TCs) represent a recently discovered unique subset of interstitial cells in mammalian (and vertebrate) tissues [10]. Presently, these cells have been observed in multiple organs, including the heart [11], lungs [12], skeletal muscle [13], pleura [14], and duodenum [15], among others. TCs have also been identified in skin [16]. TCs are interstitial and stromal cells distinguished by unique ultrastructural characteristics, including remarkably long and thin cytoplasmic extensions, termed telopodes (Tps), with alternating segments (podomers) and small dilated areas resembling cisterns (podoms), which form network patterns that define boundaries around parenchymal cell structures [17]. The number of Tps determines the morphology of the TC cell body, and alternating narrow portions and dilated portions can be used as characteristics for unambiguous recognition of TCs under transmission electron microscopy (TEM). Markers used for TC identification vary by species and tissue. However, commonly used markers for identifying TCs include CD34, tyrosine kinase receptor (c-kit), platelet-derived growth factor receptor α (PDGFR-α), and Vimentin [18,19].

In the organs studied so far, telocytes along with their podoms are capable of forming both homocellular and heterocellular junctions with surrounding tissues and neighboring cells [20], thereby forming cellular networks [21]. More significantly, telocytes are regarded as playing an essential role in intercellular communication through the connections they can establish with various cell types. For instance, the intimate proximity between TCs and mast cells has been commonly observed in human skin tissue [22]. This form of collaboration may indicate that TCs participate in either stimulating or inhibiting mast cells throughout allergic responses [23,24]. Recent research has further revealed the important role of TCs in immune regulation. For example, TCs are considered significant immune regulatory factors, and their reduction in number or functional alterations may lead to tissue inflammation [23]. In skin injury models, TCs have been validated to suppress the discharge of inflammatory mediators and facilitate epithelial cell migration, thereby engaging in the cutaneous repair process [25]. Furthermore, TCs in embryonic skin can express vascular endothelial growth factor (VEGF), suggesting their promoting role in the angiogenesis process [26].

Research demonstrates that TCs form homocellular and heterocellular contacts with surrounding epithelial cells, endothelial cells, fibroblasts, and other stromal cells through their Tps, constructing complex three-dimensional network structures [27]. In skin tissue, TEM studies reveal that TCs develop spatial three-dimensional networks, with their Tps forming heterocellular contacts with the surrounding cells—including mast cells, fibroblasts, adipocytes—as well as blood vessels, nerves, and skin appendage structures [28]. However, the overall role and mechanisms by which TCs regulate other cellular responses in chronic inflammation and fibrosis remain far from fully understood, with some studies even presenting contradictory findings. Both in vitro and in vivo cutaneous injury models have validated that skin TCs can suppress the discharge of inflammatory mediators and facilitate epithelial cell migration, demonstrating a protective function in LPS-induced cutaneous wound-healing models [29]. However, in fibrotic diseases, TC telopod networks become disrupted or disappear, and TC numbers decrease, suggesting they may exert different regulatory roles under different pathological conditions [28].

For decades, needle-free injection technologies have been applied in the delivery of medications and vaccines [30]. The majority of these devices operate on the same principle: propelling a small volume of drug- or vaccine-containing fluid to generate a fluid stream capable of directly perforating the skin and delivering the liquid into dermal tissue [31]. Needle-free injections are not only less painful than traditional needle injections, but also allow the drug to be more effectively confined to the dermis and subcutaneous fat [32]. Research has found that needle-free injection can induce potent immune responses in rats, which is primarily attributed to the more dispersed distribution pattern created by needle-free injection that increases drug contact with cells at the injection site (such as Langerhans cells, macrophages, and dendritic cells), thereby promoting the antigen presentation process [33]. Therefore, needle-free injection is a promising new immunization approach with broad prospects. However, medical scientists have always focused on the study of immune mechanisms induced by needle-free injections and less on the histological and interstitial cell population changes within the skin after needle-free injections; therefore, studies comparing the interstitial cellular kinetic changes and histological changes within the skin after normal and needle-free injections are necessary to provide assistance in the evaluation of the safety of needle-free injections and in developing more efficient transdermal drugs.

In this study, pigs were used as experimental subjects, and an inactivated porcine circovirus vaccine was delivered into the neck skin using a jet needle-free injection (JNFI) device. Untreated normal neck skin was used as the control tissue. We then examined TCs in JNFI-treated skin and in control skin specimens through histological analysis, immunohistochemistry, immunofluorescence, TEM, and TEM-based morphometry. The aim was to describe early morphological and ultrastructural changes in TCs 24 h after injection and to generate cautious morphological evidence for their possible involvement in local cutaneous responses.

## 2. Materials and Methods

### 2.1. Animals and Ethics Statement

Six piglets aged one month were sourced from Jiangsu Zhongcheng Company (Yancheng, China) and maintained at the Animal Center of Nanjing Agricultural University. The piglets underwent a one-week acclimatization period with adequate water and feed provided daily before experimentation commenced. Piglets were randomly assigned to two experimental groups, with each group containing three animals: an untreated normal-skin control group and a group receiving an inactivated porcine circovirus vaccine injection into the neck skin via the POK-MBX JNFI system. Sodium pentobarbital anesthesia (2%, 30 mg/kg) was administered before any procedures that could cause discomfort. The animals were sacrificed 24 h after injection, and neck skin tissue samples were collected. The 24 h time point was selected to capture early local inflammatory infiltration and early ultrastructural responses of interstitial cells after intradermal vaccine delivery, a period during which skin-derived immune-cell mobilization has been reported after intradermal immune stimulation. All experimental procedures were approved by the Animal Ethics Committee of Nanjing Agricultural University (protocol code: NJAU.No20240320052, date of approval: 20 March 2024).

### 2.2. HE Staining

The harvested neck skin samples were preserved by immersion in 4% paraformaldehyde for 48 h, followed by dehydration and paraffin embedding. Sections of 7 μm thickness were prepared from tissue blocks embedded in paraffin. Following deparaffinization, sections underwent hematoxylin and eosin staining, and staining outcomes were observed using an Olympus light microscope (DP73, Tokyo, Japan).

### 2.3. Toluidine Blue Staining

Mast cells in skin tissue were identified using toluidine blue staining. Skin sections (7 μm) were deparaffinized and rehydrated, then incubated with 0.5% toluidine blue solution for 15 min. Following incubation, sections were rinsed with distilled water and treated with 0.5% acetic acid until a light blue background was achieved with distinct mast cell visualization. The sections then underwent dehydration, xylene treatment, and mounting with neutral resin. Observations were made with an Olympus light microscope (DP73, Tokyo, Japan).

### 2.4. Immunohistochemistry (IHC)

Paraffin sections (7 μm) were placed on positively charged slides, underwent deparaffinization, and endogenous peroxidase activity was inhibited using 3% hydrogen peroxide, followed by antigen retrieval in citrate buffer (0.01 M, pH 6.0) at 95 °C. Subsequently, sections were blocked with 5% bovine serum albumin (BSA) and incubated overnight with rabbit anti-CD34 antibody (1:100) or mouse anti-Vimentin antibody (1:100), respectively. PBS served as a replacement for the primary antibody as the negative control. On the subsequent day, sections were washed with PBS (0.1 M, pH 7.4) and incubated for one hour at 37 °C with either HRP-conjugated anti-rabbit IgG (1:100) or HRP-conjugated anti-mouse IgG (1:100). After additional washing, peroxidase activity was detected using diaminobenzidine (DAB), followed by hematoxylin counterstaining of nuclei. IHC-positive cells were defined as cells showing a distinct brown DAB reaction product above the negative-control background and with staining localized to the expected cellular compartment. TCs were interpreted only when marker expression was combined with slender cell bodies, very long, thin processes, appropriate interstitial localization, and TEM-compatible morphology.

### 2.5. Immunofluorescence (IF)

Positively charged slides containing 7 μm paraffin sections were subjected to deparaffinization and rehydration procedures. Antigen retrieval was performed using heat-induced treatment with sodium citrate buffer (0.01 M, pH 6.0, 95 °C). After blocking tissue specimens with 5% BSA, they were incubated at 4 °C overnight with a primary antibody cocktail consisting of rabbit anti-CD34 (1:100) and mouse anti-alpha-smooth muscle actin (α-SMA; 1:100). PBS was used as a negative control instead of primary antibodies. The next day, after thorough PBS washing (0.01 M, pH 7.4) to eliminate unbound primary antibodies, specimens underwent incubation with secondary antibodies: DyLight 488-conjugated anti-mouse IgG and TRITC-conjugated anti-rabbit IgG (1:400) for 1 h at 37 °C. Nuclear visualization was achieved using 4′,6-diamidino-2-phenylindole (DAPI). An Olympus microscope (DP73, Tokyo, Japan) was employed to capture fluorescent images. IF-positive labeling was recorded when fluorescence intensity was clearly above the negative-control background and colocalized with the expected cell morphology and anatomical position.

### 2.6. Transmission Electron Microscopy (TEM)

Collected neck tissue was sectioned into 1 mm^3^ pieces and stored overnight at 4 °C in 2.5% glutaraldehyde. After rinsing with PBS (0.1 M, pH 7.4), secondary fixation was performed using osmium tetroxide (Polysciences Inc., Warrington, PA, USA) at room temperature. Following routine dehydration protocols, samples were embedded in Epon812 (Merck & Co., Inc., Rahway, NJ, USA) at 60 °C for three days. Ultrathin sections measuring 50 nm in thickness were cut and mounted on copper grids, stained with uranyl acetate and lead citrate, and subsequently observed under a Hitachi transmission electron microscope (H-7650, Tokyo, Japan).

### 2.7. TEM Morphometry and Statistical Analysis

For quantitative TEM morphometry, representative perivascular and adipose-associated fields were selected from each animal. TC profiles were identified by the combined presence of a small cell body, very long and thin Tps with moniliform dilatations, interstitial localization, and close anatomical association with vessels or adipocytes. The number of TC profiles was normalized to the analyzed TEM field area and expressed as profiles per 100 μm^2^. Visible telopod or podomere segment length was measured in micrometers, and vesicle profiles were counted per TC profile. Data are presented as mean ± SD. Comparisons between normal skin and JNFI 24 h skin were performed using an unpaired two-tailed Student’s *t*-test, and *p* < 0.05 was considered statistically significant.

## 3. Results

### 3.1. Histopathological Analysis of Normal Skin and JNFI 24 h Skin

**Figure 1:**
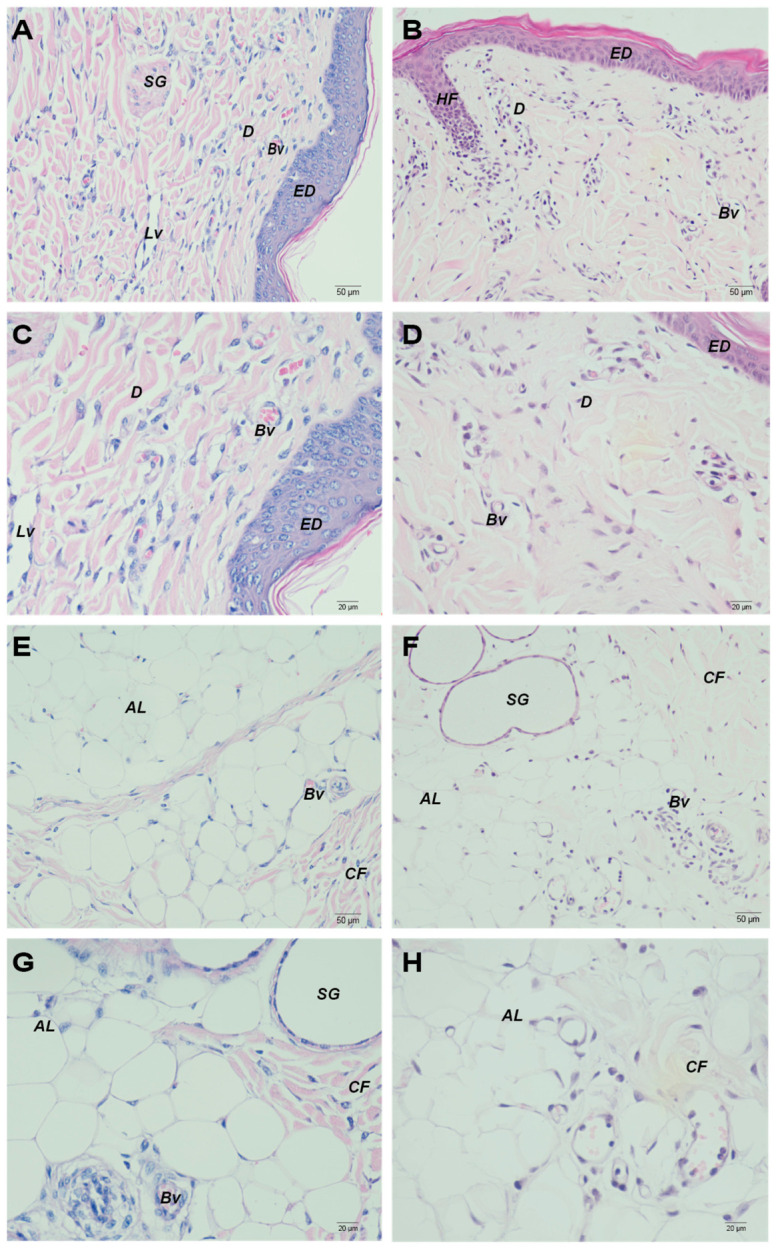
Histological images of normal skin and JNFI 24 h skin. (**A**,**C**) Histological images of the epidermis and dermis of normal skin. (**B**,**D**) Histological images of the epidermis and dermis following JNFI. (**E**,**G**) Histological images of the subcutaneous tissue of normal skin. (**F**,**H**) Histological images of the subcutaneous tissue following JNFI. ED: epidermis; D: dermis; BV: blood vessel; LV: lymphatic vessel; SG: sweat gland; HF: hair follicle; CF: collagen fiber; AL: adipose layer. Scale bars (**A**,**B**,**E**,**F**) = 50 μm; (**C**,**D**,**G**,**H**) = 20 μm.

HE staining showed that porcine skin exhibited a typical structure composed of the epidermis, dermis, and subcutaneous tissue. The epidermis was relatively thick, with a well-developed stratum corneum visible in the superficial layer (Figure 1A,C). No obvious structural damage to the epidermis was observed after JNFI. The dermis contained abundant collagen fibers, blood vessels, lymphatic vessels, and sweat glands. Following JNFI, scattered inflammatory cell infiltration was observed within the interstitial spaces between dermal collagen fibers. These cells were characterized by nearly round nuclei and a high nuclear-to-cytoplasmic ratio, with the greatest degree of infiltration observed in the papillary dermis (Figure 1B,D). The subcutaneous adipose layer in pigs was exceptionally thick, approximately 3–5 times thicker than the dermis, and contained sparsely distributed collagen fibers (Figure 1E,G). After JNFI, inflammatory cell infiltration within the collagen fibers scattered in the subcutaneous adipose tissue was more pronounced than that in the dermal collagen fibers. In contrast, no significant histological alterations were observed in the adipocytes (Figure 1F,H).

### 3.2. Identification and Spatial Distribution of TCs in Normal Porcine Skin and JNFI 24 h Skin

**Figure 2:**
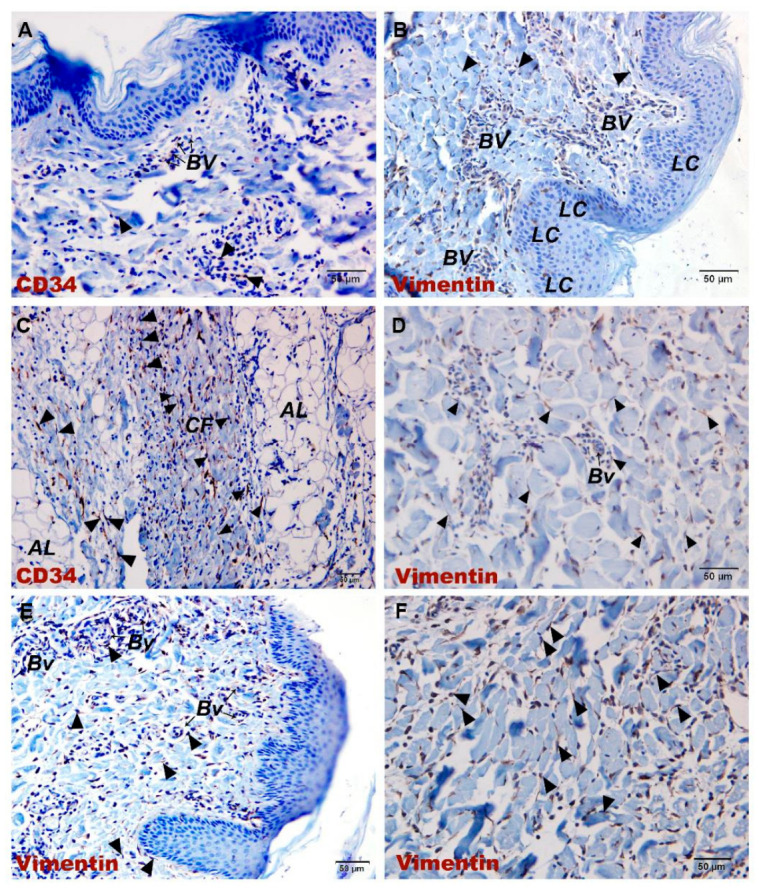
Immunohistochemical images of CD34 and Vimentin in normal skin and JNFI 24 h skin. (**A**) CD34 immunohistochemical image of the epidermis and upper dermis in JNFI 24 h skin. (**B**) Vimentin immunohistochemical image of the epidermis and upper dermis in JNFI 24 h skin. (**C**) CD34 immunohistochemical image of collagen fibers in the adipose layer after JNFI. (**D**) Vimentin immunohistochemical image of the lower dermis in JNFI 24 h skin. (**E**) Vimentin immunohistochemical image of the normal epidermis and upper dermis. (**F**) Vimentin immunohistochemical image of the lower dermis in normal skin. LC, Langerhans cells; CF, collagen fibers; AL, adipose layer; BV, blood vessel. (▲), telocyte. Scale bars (**A**–**F**) = 50 μm.

**Figure 3:**
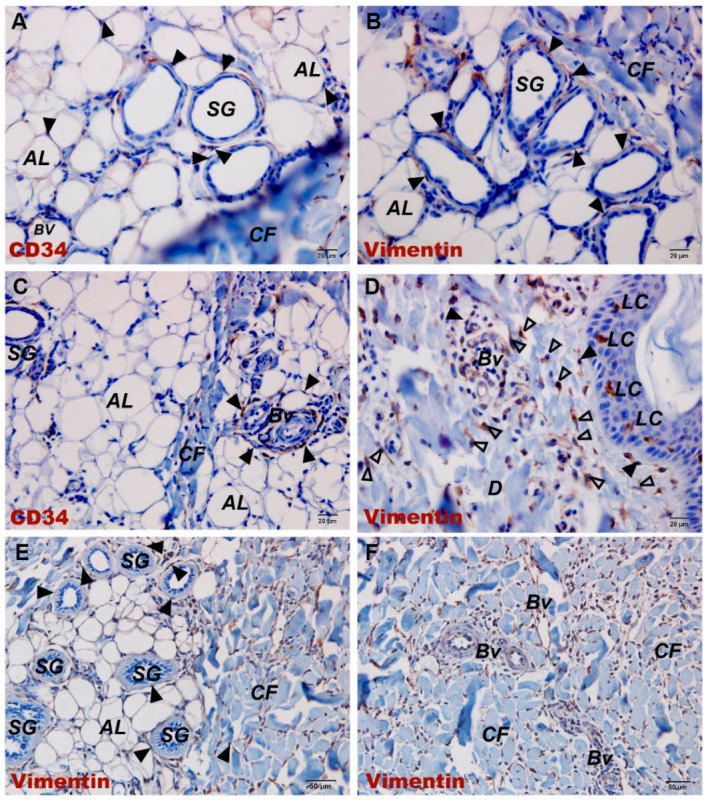
Immunohistochemical images of CD34 and Vimentin expression in TCs in normal skin and JNFI 24 h skin. (**A**) CD34 immunohistochemical image of sweat glands in JNFI 24 h skin. (**B**) Vimentin immunohistochemical image of sweat glands in JNFI 24 h skin. (**C**) CD34 immunohistochemical image of blood vessels in JNFI 24 h skin. (**D**) Vimentin immunohistochemical image of blood vessels in JNFI 24 h skin. (**E**) Vimentin immunohistochemical image of sweat glands in normal skin. (**F**) Vimentin immunohistochemical image of blood vessels in normal skin; D, dermis; AL, adipose layer; BV, blood vessel; CF, collagen fiber; SG, sweat gland. (▲), telocyte; (Δ), fibroblast. Scale bars (**A**–**D**) = 20 μm, (**E**,**F**) = 50 μm.

**Figure 4:**
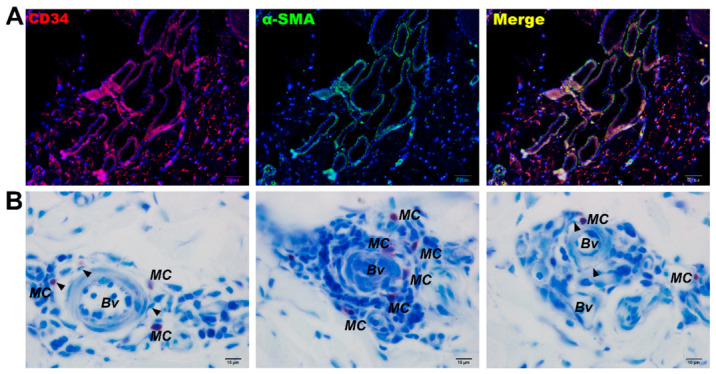
Double immunofluorescence labeling and toluidine blue staining images of sweat glands and blood vessels in normal skin. (**A**) Double immunofluorescence image of α-SMA/CD34 labeling in sweat glands of normal skin. (**B**) Toluidine blue staining image of blood vessels in normal skin. MC, mast cell; BV, blood vessel. Scale bars: (**A**) = 50 μm, (**B**) = 10 μm.

IHC and IF were used to identify the immunophenotypic markers of TCs in porcine skin. Consistent with most previous reports, porcine skin TCs expressed CD34^+^ and Vimentin^+^ and were widely distributed throughout the skin tissue, exhibiting distinct morphologies at different locations. Within the dermal collagen fibers, the cytoplasmic prolongations of TCs were arranged parallel to the basement membrane. In contrast, in the deeper dermis, their Tps encircled collagen fiber bundles and were embedded within the spaces between different collagen fibers (Figure 2E,F). Around vessel lumen-like structures and glands, TCs surrounded these structures through their extremely long and slender Tps, forming single-layered or multilayered ring-like structures (Figure 3E,F). Double IF labeling for CD34 and α-SMA revealed an unusual immunophenotype of perisudoral TCs: TCs surrounding the sweat glands were CD34^+^/α-SMA^+^, whereas TCs in the surrounding dermis were CD34^+^/α-SMA^−^ (Figure 4A). Because CD34, Vimentin, and α-SMA are not specific to TCs, these cells were identified by combining immunophenotype, long-range morphology, anatomical localization, and ultrastructural validation.

After JNFI, a large number of inflammatory cells infiltrated the dermal and subcutaneous collagen fibers. At sites of inflammatory cell infiltration, the expression of CD34 and Vimentin was markedly increased. In addition, numerous CD34^+^/Vimentin^+^ TCs with long and slender cytoplasmic processes were observed within the collagen fibers, and direct cellular contacts between TCs and inflammatory cells could be observed under the light microscope (Figure 3A–D). In fields without inflammatory cell infiltration, CD34^+^/Vimentin^+^ TCs were less prominent on qualitative examination compared with normal skin (Figure 2A–D).

### 3.3. Toluidine Blue Staining Results

Toluidine blue staining was used to identify cellular processes and mast cells in the skin. The results showed that, under steady-state conditions, the nuclei of porcine skin cells were stained blue. Around the arterioles and venules in the dermis, mast cells with metachromatic properties were observed. These cells contained abundant metachromatic granules in the cytoplasm and were therefore stained purple-red. These mast cells were mainly located outside the vascular endothelium, although some were also scattered among the collagen fibers. On the outer side of the blood vessels, extremely slender cells with blue-stained cytoplasm were seen surrounding the vessels. Under oil immersion, these cells displayed moniliform processes and were identified as telocyte-like cells. Through their long, slender prolongations, these cells formed heterocellular contacts with mast cells and perivascular cells, and they were also observed among the surrounding collagen fibers (Figure 4B).

### 3.4. Changes in the Distribution of Perivascular TCs in Normal Skin and JNFI 24 h Skin

**Figure 5:**
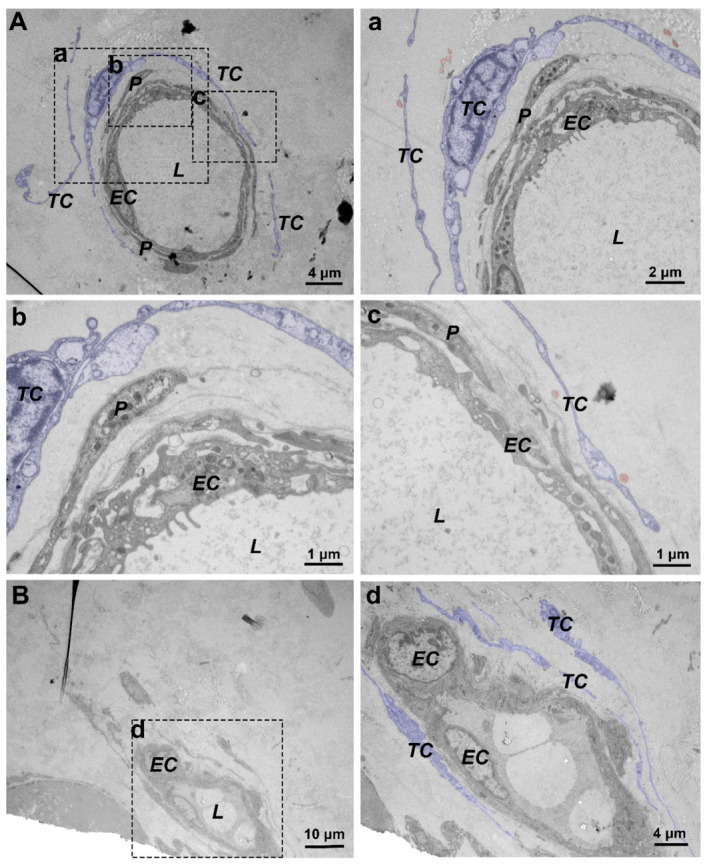
TEM images of blood vessels in normal skin and JNFI 24 h skin. (**A**) TEM image of a blood vessel in normal skin. (**B**) TEM image of a blood vessel in JNFI 24 h skin. (**a**–**c**) Higher-magnification images of selected areas in (**A**). (**d**) Higher-magnification image of a selected area in (**B**). P, pericyte; EC, endothelial cell; TC, telocyte. Scale bars: (**A**,**d**) = 4 μm; (**a**) = 2 μm; (**b**,**c**) = 1 μm; (**B**) = 10 μm.

TEM confirmed the reliability of the immunohistochemical findings and revealed the perivascular ultrastructure of the skin in different groups. In normal skin, pericytes and multiple layers of TCs surrounding the pericytes were distributed on the outer side of the capillary endothelium (Figure 5A). Pericytes exhibited a flattened and elongated morphology, whereas TCs showed a high nuclear-to-cytoplasmic ratio, and the moniliform dilatations along the Tps were clearly visible, separating the vascular structure from the surrounding collagen fibers (Figure 5(a–c)). In contrast, in the perivascular region of JNFI 24 h skin, although the perivascular arrangement of TCs was not disrupted, more vesicles released from TCs were observed among the pericytes and collagen fibers, and more unreleased vesicles were also seen within the cytoplasm of TCs (Figure 5B(d)).

### 3.5. Changes in the Distribution of Adipose-Associated TCs in Normal Skin and JNFI 24 h Skin

**Figure 6:**
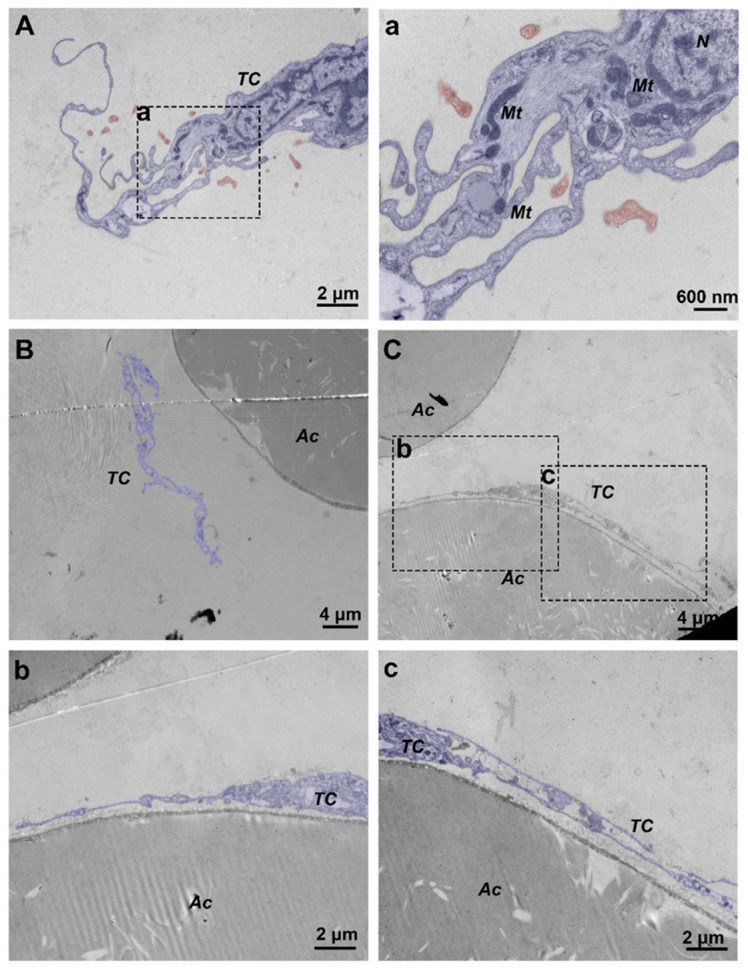
TEM images of adipose tissue in normal skin and JNFI 24 h skin. (**A**) TEM image of adipose tissue after JNFI. (**B**) TEM image of an adipocyte after JNFI. (**C**) TEM image of an adipocyte in normal skin. (**a**) Higher-magnification image of a selected area in (**A**). (**b**,**c**) Higher-magnification images of selected areas in (**C**). TC, telocyte; Ac, adipocyte; Mt, mitochondrion; N, nucleus. Scale bars: (**A**,**b**,**c**) = 2 μm; (**a**) = 600 nm; (**B**,**C**) = 4 μm.

TEM showed that a large number of adipocytes were present in the porcine subcutaneous fat layer. The cytoplasm of these cells exhibited a diffuse, homogeneous, moderately electron-dense appearance, representing the lipid storage area. Meanwhile, the nucleus was located at the cell periphery, and the plasma membrane displayed a very distinct membranous structure. In both normal skin and JNFI 24 h skin, TCs were present in the porcine subcutaneous fat layer. These cells were characterized by small nuclei and extremely long, slender processes with obvious moniliform structures, and subcellular organelles such as mitochondria and lysosomes could also be observed in their cytoplasm. In normal skin, TCs were anatomically closely associated with adipocytes, forming a ring-like cellular structure with one or more layers surrounding the adipocytes (Figure 6C(b,c)). After JNFI, the anatomical relationship between TCs and adipocytes became loosened. TCs no longer formed a ring-like cellular structure around adipocytes. Instead, they appeared in the collagen fibers outside the adipocytes, where they formed complex structural patterns through changes in their cytoplasmic morphology and released vesicles to themselves or into the surrounding environment. In the cytoplasm of TCs with this morphology, mitochondria, rough endoplasmic reticulum, and numerous unreleased vesicles could be observed, and homocellular junctions were also formed between TCs themselves (Figure 6A,B).

### 3.6. Quantitative TEM Morphometry of Perivascular and Adipose-Associated TCs

**Figure 7:**
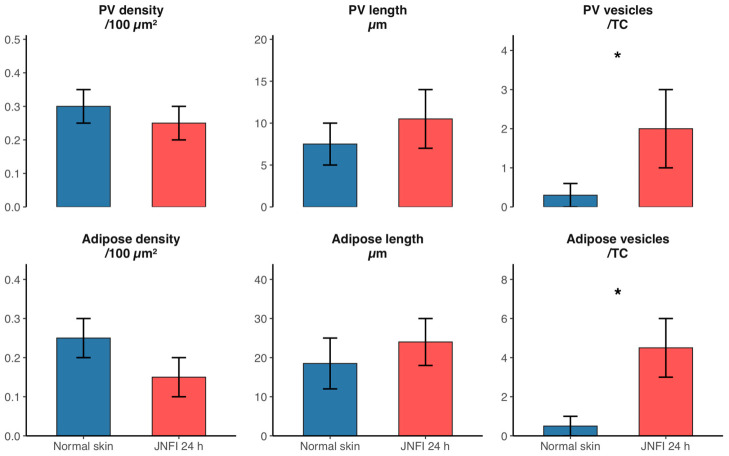
Quantitative TEM morphometry of perivascular (PV) and adipose-associated TCs in normal skin and JNFI 24 h skin. Parameters include TC profile density per 100 μm^2^, visible telopod length, and vesicle profiles per TC. Bars indicate mean ± SD. * *p* < 0.05 versus normal skin.

TEM morphometry was performed to provide quantitative support for the qualitative ultrastructural observations. In the perivascular compartment, TC profile density and visible telopod length did not differ significantly between normal skin and JNFI 24 h skin (*p* ≥ 0.05). By contrast, vesicle profiles per TC increased significantly in JNFI 24 h skin (*p* < 0.05). In the adipose-associated compartment, TC profile density and visible telopod length also did not differ significantly between normal skin and JNFI 24 h skin (*p* ≥ 0.05), whereas vesicle profiles per TC increased significantly after JNFI (*p* < 0.05). These data indicate that early JNFI-associated changes in TCs are characterized mainly by increased vesicle formation or release rather than by changes in TC profile density or visible telopod length (Figure 7).

## 4. Discussion

As an economically significant animal, the pig is an important source of meat for many countries. It is also extensively utilized as a model organism for human medicine owing to its physiological resemblance to humans. Since the pig immune system shares over 80% similarity with the human immune system, while mouse models share less than 10% similarity with humans, the importance of pig models in vaccine development and testing is increasingly prominent [34]. The epidermal-to-dermal ratio of pig skin resembles that of humans, making it particularly suitable for research on transdermal vaccine delivery [35]. Compared to traditional intramuscular injection, needle-free intradermal injection can significantly reduce stress responses in pigs, specifically manifested as marked reductions in vocalization behavior and avoidance reactions [36], while also significantly reducing carcass losses [30,37]. From physiological indicators, the serum levels of C-reactive protein and haptoglobin in the needle-free injection group were significantly lower than those in the traditional injection group, indicating less acute phase response and muscle damage [38]. Needle-free injection is now being promoted on a small scale as a route of administration for veterinary vaccines and is gaining widespread attention because of its novel injection method.

Since Spanish anatomist Cajal identified a novel interstitial cell type, interstitial cells of Cajal (ICCs), within the gastrointestinal system over 130 years ago, researchers have explored the presence of ICCs in various animals and tissues. Over time, interstitial cells exhibiting morphology comparable to ICCs have been discovered across diverse animals and organs. Yet, they remained without systematic classification until Popescu characterized some of them as a unique interstitial cell category: TCs [10]. To date, TEM remains the most precise approach for identifying TCs, although specific markers can also help distinguish TCs from other interstitial cell types.

Our results demonstrated that TCs are widely present in porcine skin, and their morphology is closely related to their distribution location. In the upper dermis, TCs were distributed in collagen fibers with extremely elongated cytoplasm parallel to the basement membrane. Meanwhile, those in the lower dermis were wrapped around and interspersed with collagen fiber bundles. TCs were also distributed around luminal structures in the skin, with one to multiple layers around blood vessels and usually only one layer around sweat glands. TCs in porcine skin expressed CD34^+^/Vimentin^+^, and TCs encircling sweat glands exhibited a specific CD34^+^/Vimentin^+^/α-SMA^+^ phenotype, whereas TCs in other sites were α-SMA^−^. In human and animal cutaneous tissue, the majority of research has validated the reliability of CD34 and Vimentin for TC identification, although the existence of α-SMA^+^ TCs in skin was previously disputed [39,40]. Multiple investigations have verified the existence of an α-SMA^+^ TC population, and in human conjunctival tissue, peripheral TCs within perivascular sheaths demonstrated intense immunoreactivity to α-SMA; correspondingly, α-SMA-positive TCs were also found in the pancreatic tissue of Chinese soft-shelled turtles [41,42].

After JNFI, clear early changes were observed within the porcine skin at the 24 h time point, including inflammatory cell infiltration and ultrastructural alterations in interstitial cells. This time point was chosen to capture early local tissue responses after intradermal vaccine delivery, but it should not be interpreted as defining the full chronological sequence of TC dynamics. TCs were able to make contact with infiltrating inflammatory cells via Tps and were found to have a specific anatomical relationship with mast cells in the perivascular area. Previous studies have suggested that TCs may contribute to the regulation of inflammatory and fibrotic tissue responses through intercellular communication [43,44]. TCs were also found to reverse lipopolysaccharide (LPS)-induced reactive oxygen species (ROS) upregulation and reduce skin wound inflammatory factor expression, supporting the possibility that TCs participate in immune modulation within the skin after JNFI [29]. Future studies should include multiple time points, such as 6 h, 12 h, 24 h, 48 h, 72 h, and later resolution or remodeling stages, to establish true chronological dynamics.

The relationship of TCs with blood vessels and adipocytes was also intriguing. In our study, TEM-based morphometry showed no statistically significant difference in TC profile density or visible telopod length between normal skin and JNFI 24 h skin in either the perivascular or adipose-associated compartment (*p* ≥ 0.05). In contrast, vesicle profiles per TC increased significantly after JNFI in both compartments (*p* < 0.05). This quantitative pattern supports the qualitative observation that the early response of TCs may be reflected more by secretory or vesicle-associated activity than by changes in cell profile density or visible telopod length. Multiple layers of TCs were present under normal conditions, both around blood vessels and around adipocytes, and they were wrapped around the associated structures by extremely elongated Tps. This surrounding structural arrangement of TCs has commonly been regarded as serving roles in mechanical stabilization and intercellular signaling, with comparable structures being identified surrounding germinal tubules, nerves, and smooth muscle [40,45,46]. The subcutaneous adipose tissue is located below the dermis, and, beyond functioning as an energy storage and thermal regulator, also houses multiple immune cell types, including T cells, B cells, and macrophages. Morphological alterations of TCs within the subcutaneous fat layer after JNFI may provide evidence of their involvement in regulating the microenvironment within the skin [3].

Following JNFI treatment, TCs relocated within collagen fibers or the adipose layer, with numerous vesicles being generated or discharged within the cytoplasm, suggesting enhanced secretory and intercellular communication activity. Multiple forms of extracellular vesicles (EVs) produced by TCs, specifically exosomes, microvesicles, and multivesicular bodies, which contain mRNA, microRNA (miRNA), long non-coding RNA (lncRNA), and proteins, have been reported to function as carriers for paracrine molecular signal transmission between TCs and other recipient cells [47,48]. However, the present study did not directly analyze EV cargo, cytokines, chemokines, porcine circovirus antigen localization, or immune-cell phenotypes; therefore, the functional interpretation should remain cautious.

Several limitations should therefore be considered. First, only six piglets were included, with three animals per group, so the findings should be interpreted as preliminary morphological evidence. Second, the control group consisted of untreated normal skin; additional PBS, adjuvant-only, and route-matched sham-injection controls will be required to distinguish the effects of mechanical jet delivery, vaccine antigen, and adjuvant. Third, only the 24 h time point was examined. Finally, although TEM provides strong ultrastructural support for TC identification, CD34, Vimentin, and α-SMA are shared with other stromal cells. Future work should combine broader marker panels, including PDGFR-α and c-kit, where suitable porcine reagents are available, with functional and molecular assays.

## 5. Conclusions

This study provides a systematic preliminary morphological description of TC changes in porcine skin under JNFI conditions. TCs were widely distributed in normal porcine skin and were located in distinct anatomical regions, including between collagen fiber bundles, around blood vessels, around sweat glands, and around adipocytes. Perisudoral TCs exhibited a CD34^+^/Vimentin^+^/α-SMA^+^ immunophenotype, whereas TCs in other regions were CD34^+^/Vimentin^+^/α-SMA^−^, suggesting possible heterogeneity among TC subpopulations within the same tissue. After JNFI, TCs showed morphological changes, including direct contact with infiltrating inflammatory cells through Tps, redistribution from ring-like structures surrounding adipocytes to surrounding collagen fibers, and a statistically significant increase in vesicle profiles in perivascular and adipose-associated compartments (*p* < 0.05). Together, these findings provide morphological evidence suggesting that TCs may participate in early skin responses triggered by JNFI and may contribute to intercellular communication through direct cell-to-cell contact and vesicle-associated signaling. Because TC identification based on CD34, Vimentin, and α-SMA is not fully specific, future studies should incorporate additional markers such as PDGFR-α and c-kit, dual or triple IF, TEM validation, larger animal cohorts, route-matched sham controls, adjuvant-only controls, porcine circovirus antigen localization, and molecular immune readouts.

## Data Availability

The original contributions presented in this study are included in the article. Further inquiries can be directed to the corresponding authors.

## Abbreviations

The following abbreviations are used in this manuscript:

BSA	bovine serum albumin
DAB	diaminobenzidine
DAPI	4′,6-diamidino-2-phenylindole
EVs	extracellular vesicles
ICC	interstitial cell of Cajal
ICLC	interstitial Cajal-like cell
IF	immunofluorescence
IHC	immunohistochemistry
LPS	lipopolysaccharide
PBS	phosphate-buffered saline
PRRs	pattern-recognition receptors
ROS	reactive oxygen species
TCs	telocytes
TEM	transmission electron microscopy
Tps	telopodes
VEGF	vascular endothelial growth factor
JNFI	jet needle-free injection
α-SMA	alpha-smooth muscle actin
